# Biobanking in Atherosclerotic Disease, Opportunities and Pitfalls

**DOI:** 10.2174/157340311795677707

**Published:** 2011-02

**Authors:** V.P.W Scholtes, J.P.P.M de Vries, L.M Catanzariti, D.P.V de Kleijn, F.L Moll, G.J de Borst, G Pasterkamp

**Affiliations:** 1 Experimental Laboratory Cardiology, University Medical Center Utrecht, Utrecht, The Netherlands; 2 Department of Vascular Surgery, St Antonius Hospital Nieuwegein, The Netherlands; 3 Department of Vascular Surgery, University Medical Center Utrecht, Utrecht, The Netherlands

**Keywords:** Atherosclerosis, novel biomarkers, multifactorial disease, pathophysiology.

## Abstract

Cardiovascular disease is the leading cause of death in Western countries and current research is still focusing on optimizing therapeutic approaches in the battle against this multifactorial disease. Concepts regarding the pathogenesis of many cardiovascular diseases originate from observations of human atherosclerotic tissue obtained from autopsies or during vascular surgery. These observations have helped us to disentangle the pathophysiology of atherosclerosis. However, identifying vulnerable patients, those prone to developing cardiovascular complications, remains difficult. The search for predictive cardiovascular biomarkers continues and large, well organized biobanks are needed to discover or validate novel biomarkers. Biobanks are an extremely valuable resource that enables us to study the influence of both genetic and environmental factors on the development of multifactorial diseases such as atherosclerosis. This review will focus on the advantages and pitfalls in atherosclerotic biobanking.

## WHY BIOBANKING?

Biobanks are organized resources of biological samples with associated clinical characteristics used for scientific investigation, they can be population or disease based [1]. The storage of tissue varies considerably, ranging from well-organized formal repositories to the informal storage of tissue specimens in a researcher’s freezer. While in the past, tissue collection was performed by local pathology departments dealing with local operating procedures, biobanks have become much more sophisticated and now work with stringent standard operating procedures (SOP’s) for tissue collection, processing and storage. In the last decade, the number of biobanks has increased significantly. In the United States (US) alone, an estimated 1 billion dollars has been invested in biobanking [2] and it has been conservatively estimated that more than 300 million tissue specimens are stored from more than 180 million cases [3]. But what are the advantages of biobanks? 

Firstly, biobanks are an important resource for identifying the causes and mechanisms of many complex diseases. Biobanks enable scientists to investigate the combined influence of genetics, life style and other environmental factors in the development of multifactorial diseases. In addition, biobanks create the possibility for scientists to collect human material prior to presentation of specific diseases and provide the ability to compare different disease stages at a molecular level. Identification of pathways involved in disease initiation or progression may lead to the discovery of new therapeutic targets but also may result in the detection of biomarkers for prediction of disease progression or outcome. Furthermore, biobanks also play an important role in the process of validation of novel discovered biomarkers.

Secondly, biobanks are highly relevant for scientists in case of rare disorders. In order to investigate rare disorders, researchers would have to wait for years to collect enough material to be able to reject or confirm their hypothesis. Therefore, a biobank consisting of human material of patients with rare disorders is valuable and will save years.

Finally, disease based biobanks are needed to validate discoveries made in animal models. Regarding cardiovascular disease, animal studies have helped us greatly to elucidate the pathophysiology of atherosclerosis. One of the most studied animals in atherosclerotic research is the Apo E knockout mouse and major steps in the understanding of cardiovascular disease have been made by these animal studies. But although these mice do develop progressive atherosclerotic lesions, plaque rupture and its consequent cardiovascular complications do not occur. Therefore, to obtain further knowledge in the process of plaque rupture and the occurrence of cardiovascular complications novel ways to investigate this multifactorial disease are needed and human atherosclerotic biobanks can play major roles in this process.

## ATHEROSCLEROTIC TISSUE BIOBANKS: A HISTORICAL REVIEW

Atherosclerotic tissue biobanks have led to the description of etiologic concepts that dominated basic research focused on disentangling the pathogenesis of atherosclerosis. Major steps in this process were taken by observations of large amounts of atherosclerotic specimens obtained from autopsy. In the late 18^th^ century, Rudolph Virchow examined atherosclerotic arteries obtained from autopsies and noticed cellular inflammatory changes in the atherosclerotic vessel wall and introduced the term endarteritis deformans [4]. It was one of the first in-depth studies to focus on histological characteristics of the atherosclerotic lesion. One century later, Russell Ross combined histological observations in atherosclerotic specimens with *in vivo* experiments [5] and postulated his famous response to injury theory [6]. Michael Davies was one of the pioneers of thrombus formation, he defined three stages of thrombus evolution due to plaque rupture: intraplaque thrombus, transitional or mural thrombus, and occlusive thrombus [7]. He also provided an excellent overview of previous histological studies that demonstrated plaque vulnerability to be a function of increased numbers of macrophages, reduced smooth muscles and a large lipid core with a thin fibrous cap [8-11]. Dr. Virmani’s group investigated acute coronary artery thrombosis in patients with sudden cardiac death and found out that the majority was caused by plaque rupture (60%), but also plaque erosion (35%) and finally calcified noduli contributed to arterial thrombosis (5%) [12]. These studies are all examples of how observations of large tissue cohorts have helped unravel the pathophysiology of atherosclerosis. Hurks and colleagues published a detailed overview of the history of atherosclerotic biobanking [13]. 

## THE VULNERABLE PLAQUE

The vulnerable plaque, prone to rupture, is characterized by its large lipid core covered by a thin fibrous cap, large macrophage infiltration and low smooth muscle cell content. The aforementioned studies all contributed to the vulnerable atherosclerotic plaque paradigm. Overviews and histological classifications of the vulnerable plaque have been published over the years [14-16] and multiple definitions have been documented. Detection of these lesions before rupture is difficult although progress has been made. A recent review summarized several newly developed imaging modalities to detect the so called thin cap fibro-atheroma (TCFA), the precursor of ruptured plaques [14]. However, all of these imaging modalities have the major limitation that only a certain part of the vascular tree can be visualized. Since atherosclerosis is a systemic disorder one can only speculate where to look for the so called vulnerable plaques. 

The vulnerable plaque concept carries an important limitation which is an inherent drawback of most tissue biobank studies—it is based on cross-sectional and retrospective studies on ruptured plaques. Robust prospective, longitudinal studies on vulnerable plaque characteristics and outcome are lacking. In addition, hallmarks of the vulnerable plaque, inflammation and a large lipid core, can also be observed in asymptomatic patients and can be considered as a common and locally observed phenomena of the whole vascular tree [17]. Also, it is important to realize that not all ruptured plaques will cause arterial thrombosis. Silent rupture with plaque progression occurs [18] and 20% of asymptomatic carotid arteries show signs of plaque rupture at postmortem examination [19]. It is therefore questionable if detection of precursor lesions of the vulnerable plaque will result in identification of those patients that will develop a plaque rupture. Because of these limitations, novel strategies are being developed to detect patients at increased risk for plaque rupture. Naghavi *et al* introduced the term ‘vulnerable patient’ in 2003 [20]. They reported that the clinical presentation of a plaque rupture is influenced not only by plaque vulnerability, but also by thrombogenic blood (vulnerable blood), and electrical instability of the myocardium (vulnerable heart). The quest for identification of this vulnerable patient resulted in many large scale population studies, trying to find the holy grail that is being able to detect those patients at increased risk for cardiovascular events. Thus, although the concept of the vulnerable plaque resulted in important insights in pathogenesis of the disease, it may not optimally serve as a basis for prediction of the patient at risk of suffering a cardiovascular event. 

## POPULATION BASED BIOBANKS AND THE SEARCH FOR PREDICTIVE BIOMARKERS IN CVD

One of the first and famous examples of a population based biobank was the Framingham Heart Study. In 1951, 28,000 citizens from the town Framingham (US) were enrolled and periodically monitored. The occurrence of cardiovascular events was correlated with data from physical examinations, questionnaires and laboratory tests [21]. The Framingham Heart Study gave rise to many novel insights in the pathophysiology of cardiovascular disease and ultimately resulted in the Framingham Heart risk score for risk stratification of cardiovascular disease. It is an excellent example of a large scale study that is able to reveal factors that influence multifactorial diseases. 

In order to identify the vulnerable patient, several large scale population based biobanks have attempted to find novel serum biomarkers to predict cardiovascular risk more accurately than traditional risk factors. Several biomarkers have been found to be strongly associated with future cardiovascular events but on top of the traditional risk factors their extra predictive value was limited [22-26]. Even the addition of multi-marker panels on top of traditional risk factors has so far resulted in no or small increases in the discriminative power to detect patients at risk [27-29]. Zethelius showed improved discriminative power (the so called C-statistics,) when 4 markers (troponin I, N-terminal pro-brain natriuretic peptide, cystatin C and C-reactive protein) were added to the traditional risk factor model in 1135 elderly men [30]. However, more work is needed before multimarker panels can provide a basis for prognostic evaluation of the individual patient [31]. 

## OPPORTUNITIES IN BIOBANKING

Cross-sectional atherosclerotic biobanks may help us to generate hypotheses regarding the mechanisms of atherogenesis and its progression. However, for prediction (longitudinal) studies follow up is required, therefore, atherosclerotic biobanks with a longitudinal study design might facilitate the discovery of biomarkers that are predictive for future cardiovascular events. Taking into account that atherosclerosis has been considered as a systemic disease [32] and that plaque composition corresponds between different arterial segments [33], one could speculate that local plaque characteristics represent plaque progression in other territories of the vascular tree. Therefore, atherosclerotic plaques, obtained during vascular surgery, might contain markers predictive for future cardiovascular events. One example of this concept is the Athero-express study, which is an ongoing prospective cohort study started in 2002 [34]. The Athero-express study, of more than 1500 patients, correlates characteristics of dissected plaques with secondary cardiovascular manifestations acquired during a three year follow-up. Hellings *et al* showed that plaque composition is an independent predictor of restenosis after carotid endarterectomy. They demonstrated that patients with more stable plaque phenomena had an increased risk for the development of restenosis compared with patients with unstable plaque phenomena [35]. This was the first example that atherosclerotic plaques contain predictive information about outcome following surgery. Recently de Kleijn *et al* demonstrated that local plaque proteins are a source of biomarkers with strong predictive value for future cardiovascular events [36]. Analysis of the proteome of atherosclerotic plaques, from patients with and without secondary cardiovascular manifestations, revealed osteopontin (OPN) as a potential predictive marker. Validation of this marker in a large cohort demonstrated that patients within the highest quartile of plaque OPN levels had a 4 fold increased risk for secondary cardiovascular events compared with patients in the lowest quartile of OPN [36]. Thus, the OPN levels in one small dissected plaque revealed information regarding the risk of secondary manifestations during 3 years follow up. Unfortunately, these predictive markers are measured in surgically obtained specimens and these snapshots are not able to monitor the effect of treatment or intervention, therefore the clinical utility of predictive plaque markers is limited. 

While established circulating biomarkers are, on top of established risk factors, limited in the prediction of future cardiovascular events, the search for identification of the vulnerable patient continues. One example of a novel strategy is the Circulating Cells project. The Centre of Translational Molecular Medicine (CTMM), a consortium of 5 Dutch academic centers collaborating with industrial partners, started this multicenter study in 2009 [37]. In this study, blood will be obtained from at least 500 patients prior to percutaneous coronary intervention (PCI). Activation markers and responsiveness of the circulating cells will be measured and correlated with future cardiovascular events. Different cell fractions will be stored and used for detection of novel biomarkers by using a proteomic approach to compare the proteome of patients with and without a cardiovascular event. Hopefully novel predictive biomarkers will be found and, after validation, used in a clinical setting to detect those patients at increased risk. 

Another opportunity is the rapidly growing field of genomics. Sequencing of the human genome continues and large population based biobanks enable scientists to investigate the effect of the genomic variability on cardiovascular disease. A lot of attention has been received by the Genome Wide Association (GWA) studies in which allelic frequency variations in single nucleotide polymorphisms (SNP’s) are compared between patients with or without cardiovascular manifestations [38]. These GWA studies had a major boost in 2007 when 4 independent large scaled studies reported that the same SNP, located on chromosome 9p21.3, was associated with coronary heart disease and myocardial infarction and confirmed an increased risk predisposition [39-42]. Knowledge of the genetic predisposition for cardiovascular disease may improve clinical management and translation might be effected by two principal routes. Firstly, the discovery of regions of the genome that are associated with disease will reveal novel causal pathways and identify potential new therapeutic targets. Secondly, the knowledge of individual patterns of disease predisposition can be used to develop a more personalized disease management. In the future, novel susceptibility loci will be discovered and this information will shed light on the complex relationships between changes in the genome and disease phenotype. 

With the increasing understanding in the pathogenesis of atherosclerosis, substantial progression has been made in the development of novel imaging techniques to detect the so called vulnerable plaque. Due to a lack of a golden standard, atherosclerotic biobanks play important roles in the validation process of these novel imaging technologies. Magnetic resonance imaging (MRI) has emerged as a promising novel technique with its superior capability to determine plaque size and composition (regarding lipid-rich necrotic core and intraplaque haemorrhage) and with a high intra-reader, inter-reader and inter-scan reproducibility [43]. High resolution MRI scans have also been combined with novel tracer techniques. One example is the use of ultra-small super paramagnetic particles of iron oxide that are able to accumulate passively in plaque macrophages and therefore can be used to visualize plaque macrophages [44]. Molecular MRI is another promising field where antibodies against oxidized-LDL, Vascular Cel Adhesion Molecule-1 (VCAM-1) and Matrix Metallo Proteases (MMP’s) are linked with radiotracers and can be used to visualize different atherosclerotic molecular markers. In the near future, clinical implementation of plaque imaging techniques is likely to occur and will enable physicians not only to detect vulnerable patients but also monitor the effect of different treatment modalities. 

## CHALLENGES AND PITFALLS

With the increasing number of biobanks and increasing opportunities to store different cell fractions some challenges and pitfalls merit careful consideration. While Rudolph Virchow drew conclusions from autopsy specimens obtained from a limited number of patients, research now may include thousands of patients. Enormous databases are needed to store the data obtained from long questionnaires, cytokine profiles, genomics and proteomics. Thus, presenting researchers with a challenge to work effectively and efficiently with such datasets. Fortunately, novel software and analytical programs are available. Large online databases like online mendelian inheritance in man (OMIM^®^) and ingenuity pathway analysis (IPA^®^) provide useful tools to find novel pathways and collaborate with other scientists. 

There is a need for the simultaneous assessment of multiple markers. Availability of tissue and blood may be limited, especially of patients who are defined as a “case”. Therefore techniques have been developed that enable quantitative assessment of many markers simultaneously and require limited sample volumes. An example of a now frequently used tool, is the multiplex immunoassay technology (MIA). Cytokines reflect local or systemic inflammation and might therefore be a suitable target in the continuing quest for novel biomarkers. While enzyme linked immunoassays (ELISA) can only detect one cytokine in one sample, MIA enables detection of multiple cytokines, chemokines and other proteins in one single sample and therefore prevents spoiling large amounts of valuable and often limited human blood. 

Recent studies raised a serious concern regarding storage of samples in biobanking. Large population biobanks, but also large conducted randomized clinical trials frequently measure proteins at different points in time using frozen samples. However, sampling, handling and storage can greatly influence the reproducibility and reliability of these immunoassays. De Jager *et al* demonstrated that the type of anticoagulant used can influence the levels of several cytokines [45]. In addition, they demonstrated that most cytokines are stable for up to two years of storage but degrade after that, with most cytokines reaching almost 25% of its original value after two years [45]. They emphasized the importance of internal controls and quality checks.

Biobanks and their relevant medical ethics committees carry a serious responsibility to the patients from whom they collect their samples. Legislation of privacy issues in cardiovascular biobanking is well organized for some issues such as that patients’ data must be anonymously processed. But it is also important to ask patients, before admission to the study, what to do in case of discovery of novel beneficial or adverse biomarkers. Patients should be well informed about the possibilities in case of such discoveries. Secondly, it is important to define who owns the tissue in case of long term storage. As the number of biobanks increases so does the number of private companies offering to store cells. One of the first examples of commercialised biobanking interfering with concerns about ownership and privacy issues was the Iceland Biogenetic Project. In this project the involvement of deCODE genetics, a commercial company with monopoly rights over scientific discoveries generated within the project, generated concerns about the commercialisation of the material and sensitive personal information that was being collected without the explicit informed consent of individuals [46]. These concerns were broadly discussed in the media and finally resulted in new legislation. From now on all citizens in Iceland are included in this bank unless they file a special opt-out request form. Another example of the need for well-defined privacy legislation was provided by a case in Sweden when the government bypassed informed consent rules to gain access to an anonymous sample, stored in a Swedish biobank, of a 25 year old, whose DNA matched with DNA found on the knife that was used to kill Swedish foreign minister Anna Lindh. Examples such as these may fuel the growing criticism of the privacy policies of biobanks. The European Union legislation concerning ownership is difficult and differs per country. In addition there are huge differences between European countries in terms of government involvement in biobanks. In some Scandinavian countries governments play major roles in the development of national biobanks [47], while in other countries government involvement is very limited. With increasing international collaboration efforts should be made to standardise legislation. 

**Opportunities in Biobanking**Atherosclerotic tissue banks with a longitudinal study design: Local plaque proteins are a source for biomarkers with strong predictive value for future cardiovascular events.The quest for novel biomarkers: Correlate cardiovascular outcome with the expression of activation markers and responsiveness of the circulating cells from patients with coronary artery atherosclerosis.Genome Wide Association (GWA) studies Variations in single nucleotide polymorphisms (SNP’s) are compared between patients with or without cardiovascular manifestations and will reveal novel causal pathways and identify potential new therapeutic targets.Novel imaging techniques Progression has been made in detecting the so called vulnerable plaque. Some techniques already are at the precipice of translation to clinical practice.

**Challenges and pitfalls in Biobanking**Deal with enormous datasets: Large online databases like OMIM^®^ and Ingenuity^®^ pathway analysis provide useful tools to find novel pathways.Attention towards trivial processes: Sampling, handling and storage can greatly influence the reproducibility and reliability of immunoassays. **Legislation concerning privacy issues and ownership: ** Unified worldwide legislation regarding privacy issues and ownership is needed.**Registration of biobanks** Worldwide obligatory registration of biobanking is necessary

The third issue concerns registration. While it is obligatory for randomized clinical trials to register before starting, registration for biobanks is not mandatory. In 1999, the National Bioethics Advisory Commission of the US started an inventory about the amount of stored tissue in the United States, which resulted in the handbook of human tissue sources [3]. This was the first inventory of stored tissue in the US. In Europe a project was recently started by the European union, that enables biobanks to register in order to share data [1] which will hopefully facilitate future collaboration. 

In conclusion, biobanking of human tissue and blood samples is a hot topic. Biobanks enable us to investigate the complex interplay between genetics, environmental factors and the pathophysiological features of multifactorial diseases. Important steps in the understanding of the pathophysiology of atherosclerosis have been made by observations from large cohorts of atherosclerotic specimens. Although the vulnerable plaque paradigm has some limitations, efforts have been made to develop novel imaging modalities to identify pre-cursor lesions and identify the vulnerable patient. On the other hand, the identification of the vulnerable patient continues with the assessment of multiple systemic biomarkers in the circulation in large scaled population studies. Until now, this identification process has not resulted in the golden bullet to identify the vulnerable patient. New opportunities in this field lie in the discovery of novel, more predictive biomarkers, in atherosclerotic tissue, but also in the more easy accessible circulation. Genome sequencing and genome-wide association studies will reveal more about the influence of genetics on atherosclerosis and hopefully will discover novel therapeutic targets. A lot of progress has been made in the development of novel imaging techniques to visualize unstable plaques and even some techniques are close to translation to the clinical practice. Pitfalls include trivial processes like handling and storage of patient material. Challenges lie in collaboration of biobanks and world-wide legislation and more important, registration of biobanks.
